# Supplementary material for: Effects of dual-task interference on swallowing in healthy aging adults
Source: PLoS One. 2021 Jun 24;16(6):e0253550. doi: 10.1371/journal.pone.0253550 (PMC8224877; doi:10.1371/journal.pone.0253550)
Supplement: Attachment — Submitted filename: Response to reviewers.docx [file pone.0253550.s002.docx]

May 24, 2021

Dear Dr. Petri:

I am submitting a revised version of the manuscript PONE-D-21-01457: Effects of dual-task interference on swallowing in healthy aging adults. I would like to thank you and the reviewers: Drs. Santarcangelo and Garner for the thoughtful review of the manuscript and invaluable suggestions to improve it. We have incorporated the suggested changes into the manuscript. Please see our responses to each suggestion below:

**Editorial comments**

| **Sl. No** | **Editor comments** | **Action taken** |
| --- | --- | --- |
| 1. | Please ensure that your manuscript meets PLOS ONE's style requirements, including those for file naming. | The manuscript has been revised according to PLOS ONE's style requirements. |
| 2. | Please provide additional details regarding participant consent. In the ethics statement in the Methods and online submission information, please ensure that you have specified (1) whether consent was informed and (2) what type you obtained (for instance, written or verbal, and if verbal, how it was documented and witnessed). If your study included minors, state whether you obtained consent from parents or guardians. If the need for consent was waived by the ethics committee, please include this information. | Has been reported in the methods section.  Page 7; Lines - 139 – 141. |
| 3. | In line with PLOS' guidelines on detailed reporting (https://journals.plos.org/plosone/s/criteria-for-publication#loc-3), please ensure that you have provided sufficient detail in the Methods section on how and from where younger adult participants were recruited. | Has been reported in the methods section. Page 7; Line 130. |
| 4. | Please improve statistical reporting and report exact p-values for all values greater than or equal to 0.001. | Exact p values have been reported. |
| 5. | We note that you have indicated that data from this study are available upon request. PLOS only allows data to be available upon request if there are legal or ethical restrictions on sharing data publicly. | Yes, we will submit the raw data. |

**Reviewer Comments:**

| **Sl. No** | **Reviewers comments** | **Action taken** |
| --- | --- | --- |
|  | **Interpretation**  The authors do well to clarify how it is important to not overreach conclusions regarding hemispheric dominance based on behavioural data alone. I think that the following interpretations should at least be considered in the discussion:  i) is it certain that the finding that older adults did not show a significant interaction on the volume per time measure of swallowing behaviour due to a floor effect? The VPT for older adults looks substantially lower than that for younger adults, and so I wonder what the range on this measure actually is - i.e. is the measure sensitive/appropriate to pick up interference in older adults? | We thank the reviewer for this observation. However, we respectfully disagree on the possibility of a floor effect. The values for VPT in the current study are well within the normative range for healthy older adults.  Mean (SD): 14.68 (6.11).  We have acknowledged that volume related measures (VPT, and VPS) may be immune to dual tasking effects.  Pg. No – 14; Lines 282-284 |
|  | the interference task did not require an overt response. Therefore, could it be that older adults expressed greater performance decrements on the task for which there are no behavioural measurements? | We have added this caveat in the discussion section. |
|  | **Results**  The results are clear but some critical details are missing that would inform interpretation and enable future meta-analyses - specifically, could the authors present attained p-values, effect sizes, and confidence intervals on those effect sizes? | Exact p values have been reported.  Effect sizes: Cohen’s *d* has been reported for comparisons with statistical significance. |
|  | **Re:**  the figures - it is unclear what the error bars are depicting. Could the authors amend the figure captions to make this clear?  Figures 1-3 seem unnecessary, as the data are presented again in Figures 5-6. | Figures 1 – 3 have been removed.  Description for the figures have been provided. |
|  | **Introduction**  From the introduction on hemispheric specialization on swallowing, I was unclear exactly what the fMRI and PET data suggest re: hemispheric dominance - i.e. is it the left or the right hemisphere? Is the literature convergent in the findings? | Changes have been made to the statement. Pg. No – 2; Lines 35-38 |
|  | The Daniels et al [27] paper seems particularly important for the study. It would be good to be able to understand how those specific conclusions were reached re: left vs right dominance for volume and temporal parameters of swallowing. | Changes have been made to the statement. Pg. No – 3; Lines 61-64 |
|  | Re: the Malandraki et al [14] study - it is not clear what was found for the older adults and thus what could be concluded.  I am guessing that there were no hemispheric differences in older adults, but was there a statistically significant age-group x laterality interaction? | Changes have been made to the statement. Pg. No – 4; Lines 84-87 |

Thanks again for your conscientious review with this manuscript. We look forward to hearing more about the review of this resubmission.

Sincerely,

Balaji Rangarathnam
